# Total pancreatectomy in a patient with a dermadrome caused by intraductal papillary mucinous neoplasm: A case report

**DOI:** 10.1016/j.ijscr.2024.109645

**Published:** 2024-04-21

**Authors:** Masashi Kuno, Katsutoshi Murase, Masahiro Fukada, Yuta Sato, Jesse Yu Tajima, Nobuhisa Matsuhashi

**Affiliations:** Department of Gastroenterological Surgery and Pediatric Surgery, Gifu University Graduate School of Medicine, 1-1 Yanagido, Gifu City 501-1194, Japan

**Keywords:** Dermadrome, Intraductal papillary mucinous neoplasm, Total pancreatectomy

## Abstract

**Introduction:**

Dermadrome is a term coined by combining the words “dermatology” and “syndrome,” and it refers to dermatological symptoms that reflect visceral lesions.

**Presentation of case:**

Herein, we present the case of an 83-year-old female patient who presented with generalized blistering and erythema during treatment for acute pancreatitis. She was referred to our dermatology department with worsening erythema, although the acute pancreatitis improved. The cause of the erythema was suspected to be drug-induced, infectious, or related to collagen disease; however, the exact cause was unknown. Computed tomography and endoscopic ultrasonography findings revealed a mixed-type intraductal papillary mucinous neoplasm (IPMN). Refractory erythema was suspected to have been caused by a dermadrome due to IPMN. Consequently, she was referred to our department. The main pancreatic duct was dilated along its entire length, and tumor extension was difficult to determine; therefore, a total pancreatectomy was performed. The postoperative course was uneventful, and erythema gradually improved. The histopathological evaluation indicated high-grade dysplasia of the IPMN.

**Discussion:**

The patient's skin rash, which did not improve with treatment that included high-dose steroids, began to improve after surgery, and the disease was thought to be a dermadrome caused by IPMN.

**Conclusion:**

We believe that this is the first reported case of IPMN with a dermadrome that resolved after a total pancreatectomy.

## Highlights

• An 83-year-old female patient presented with generalized blistering and erythema.
• The patient's skin rash improved after surgery.
• The disease was identified as a dermadrome caused by IPMN.
• This is the first reported case of IPMN with a dermadrome that resolved after a total pancreatectomy.

## 1 Introduction

The term “dermadrome” was coined by combining the words “dermatology” and “syndrome,” and refers to dermatological symptoms that reflect visceral lesions. Various dermatological symptoms, including dermadromes, are caused by visceral tumors. Previous reports have suggested improvements in skin lesions after tumor excision. In this report, we describe a case of a dermadrome associated with intraductal papillary mucinous neoplasm (IPMN) that resolved after a total pancreatectomy, along with a literature review.

This case was presented in accordance with the updated consensus Surgical Case Report (SCARE) 2020 guidelines [1].

## 2 Presentation of case

**Fig. 1:**
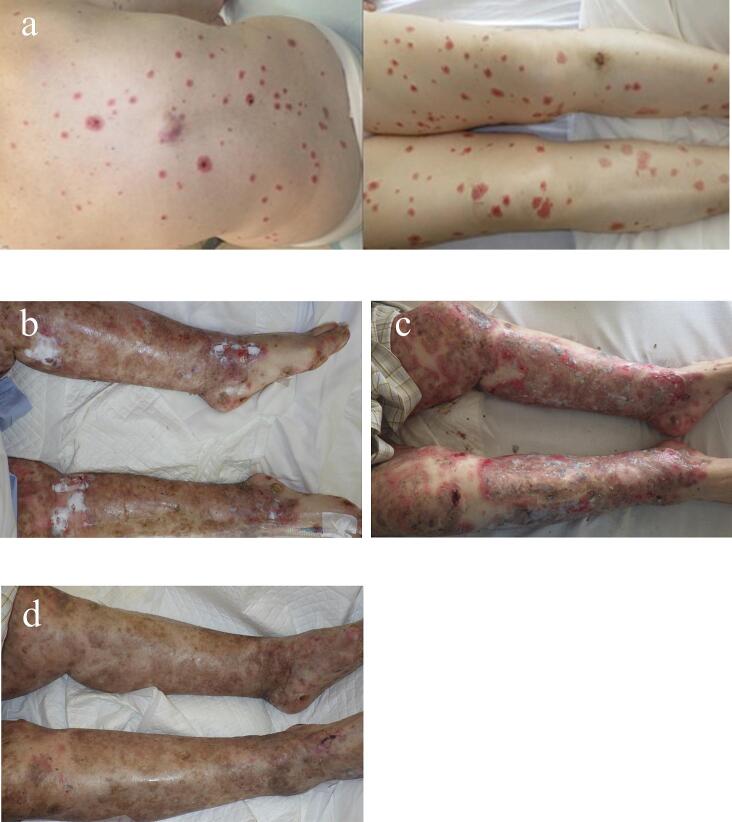
a: Upon presentation to our hospital, erythema, blisters, and erosions had spread to the extremities and trunk. b: Three days prior to surgery, despite receiving steroid treatment, the erythema continued to worsen, accompanied by erosions around the affected red areas. c: One week after surgery, pigmentation is present, but erosions are undergoing epithelialization, and no new blisters are observed. d: One month after surgery, erosions are completely epithelialized, and only pigmentation is present. (For interpretation of the references to colour in this figure legend, the reader is referred to the web version of this article.)

An 82-year-old female patient was admitted to a different hospital with acute pancreatitis. During acute pancreatitis treatment, erythema appeared throughout her body. Although the acute pancreatitis improved, the rash gradually worsened, and the patient was referred to our hospital. On presentation, the patient had multiple erythematous blisters and erythema over a wide area of the extremities and trunk (Fig. 1a). Hematological examination revealed no inflammatory response; all tumor and collagen disease-related markers, such as antinuclear antibodies, were within normal limits. No deficiencies in trace elements such as zinc or nutrients, such as essential fatty acids, were noted. Glucagon levels were also within normal limits (Table 1). Two skin biopsies were performed; autoimmune bullous diseases, such as pemphigus, were negative. The subcutaneous adipose tissue showed no abnormalities.

**Table 1:** Preoperative laboratory data.

Preoperative laboratory data			
WBC	5740/mm^3^	BUN	7.4 mg/dl
Hb	10.3 g/dl	CRE	0.87 mg/dl
Plt	18.0 × 10^4^ mm^3^		
		CEA	3.6 ng/ml
TP	6.7 g/dl	CA19–9	<2.0 U/ml
ALB	3.8 g/dl	CA125	19.8 U/ml
AST	27 IU/l		
ALT	25 IU/l	ANA	(−)
ALP	73 u/l	Anti-Dsg1Ab	<3.0 U/ml
γ-GTP	19 IU/l	Anti-Dsg3Ab	<3.0 U/ml
T-Bil	0.5 mg/dl	Anti-BP180Ab	<3.0 U/ml
Na	141 mEq/l		
K	4.1 mEq/l	CRP	0.43 mg/dl
Cl	108 mEq/l		
		Glucagon	36.5 pg/ml
Cu	115 μg/l	GLU	144 mg/dl
Zn	68 μg/dl	HbA1c	7,1 %
VitB12	1340 pg/ml		
EPA	49.5 μg/ml		

γ-GTP: γ-glutamil transpeptidase, ALB: albumin, ALP: alkaline phosphatase, ALT: alanine aminotransferase, ANA: anti-nuclear antibody, Anti-BP180Ab: anti-BP180-NC16a antibody, Anti-Dsg1Ab: anti-desmoglein 1 antibody, Anti-Dsg3Ab: anti-desmoglein 3 antibody, Anti-Sm Ab: anti-smith antibody, AST: aspartate aminotransferase, BUN: blood–urea–nitrogen, C3: complement C3, C4: complement C4, CA125: carbohydrate antigen 125, CA19–9: carbohydrate antigen 19–9, CEA: carcinoembryonic antigen, CRE: creatinine, CRP: C-reactive protein, DHA: docosahexaenoic Acid, DHLA: dihomo-gamma-linolenic Acid, ds-DNA IgG: anti-double stranded DNA immunoglobulin G antibody, EPA: eicosapentaenoic Acid, GLU: glucose, Hb: hemoglobin, HbA1c: hemoglobin A1c, HSV IgM: herpes simplex virus immunoglobulin M, IgA: immunoglobulin A, IgE: immunoglobulin E, IgG: immunoglobulin G, IgM: immunoglobulin M, LDH: lactate dehydrogenase, Plt: platelets, SCC: squamous cell carcinoma antigen, RF: rheumatoid factor, T-Bil: total bilirubin, TP: total protein, VitB12: vitamin B12, WBC: white blood cells.

No obvious source of infection was identified, and it was subsequently ruled out. All medications were discontinued or changed after admission; however, no improvement was observed, and drug-related complications were ruled out. The symptoms and blood test results were negative for collagen diseases. Finally, an evaluation for systemic neoplastic diseases was performed to investigate the possibility of tumor-related diseases.

**Fig. 2:**
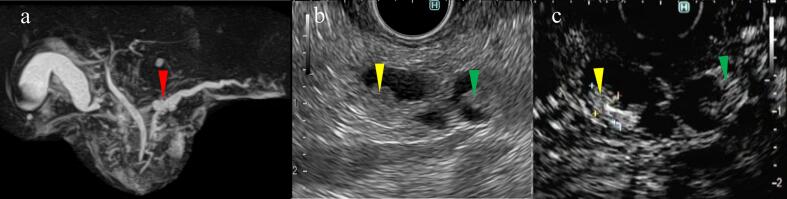
a: Magnetic resonance imaging findings reveal complete main pancreatic duct dilatation, and a cystic lesion (red arrowhead) with a septal wall is situated in the cephalic transition region, making direct contact with the main pancreatic duct. b: Endoscopic ultrasonography findings showing nodules measuring 6 mm in the main pancreatic duct (yellow arrowhead) and a cyst (green arrowhead). c: The nodules (arrowheads) were contrast-enhanced with Sonazoid. (For interpretation of the references to colour in this figure legend, the reader is referred to the web version of this article.)

Upper and lower gastrointestinal endoscopy revealed no abnormalities. Whole-body computed tomography revealed main pancreatic duct dilatation throughout the pancreas and a multifocal cystic mass in the pancreatic body. Abdominal magnetic resonance imaging (MRI) findings revealed main pancreatic duct dilatation throughout the pancreas and a cystic lesion with a septal wall in the pancreatic head–body transition area (Fig. 2a).

Endoscopic ultrasonography (EUS) findings revealed a 6-mm mural nodule in the main pancreatic duct and a cyst (Fig. 2b). Sonazoid demonstrated a contrast effect (Fig. 2c), suggesting a mixed-type IPMN with high-risk stigmata. Topical treatment for erosions, steroids, immunosuppressive drugs, nutritional supplementation, and IgG replacement therapy was initiated, but the rash worsened (Fig. 1b). An oral rash also appeared, which made oral intake difficult, resulting in physical and mental exhaustion.

After ruling out drug, infectious, and autoimmune causes, the patient was referred to our department to undergo surgery for a dermadrome associated with an IPMN. Intraoperative findings suggested that the pancreatic head was sclerotic due to pancreatitis. Consequently, a total pancreatectomy was performed.

**Fig. 3:**
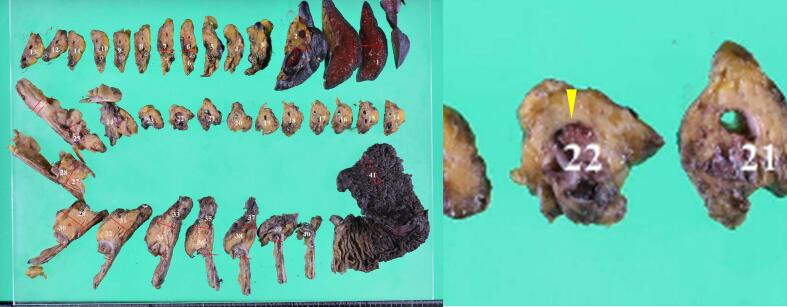
Images of the resected specimen showing a 4-mm papillary tumor in the main pancreatic duct in the body of the pancreas (arrowhead).

**Fig. 4:**
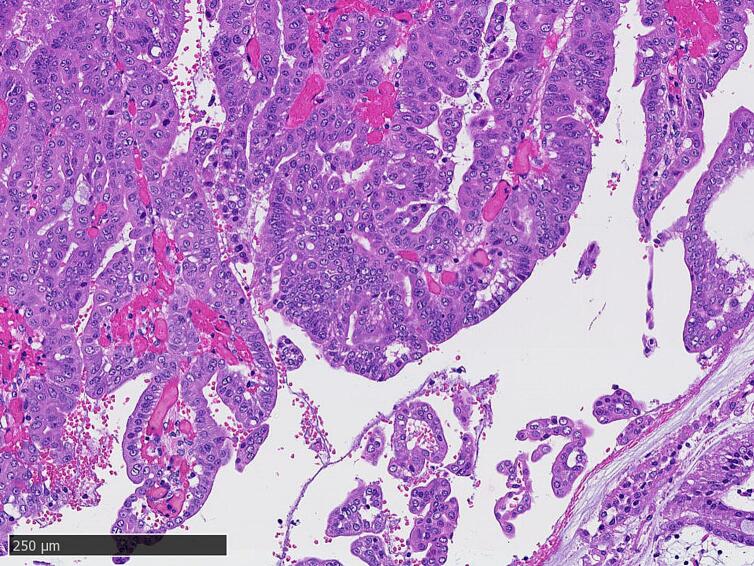
Photomicrograph of the resected specimen showing intraductal papillary mucinous neoplasm in the pancreatic duct without invasion. Classification of Pancreatic Carcinoma 7th Pb, TS1 (4 mm), intraductal papillary mucinous neoplasm, noninvasive, pTis, pBCM0, pDPM0, pN0, pStage0, R0.

**Fig. 5:**
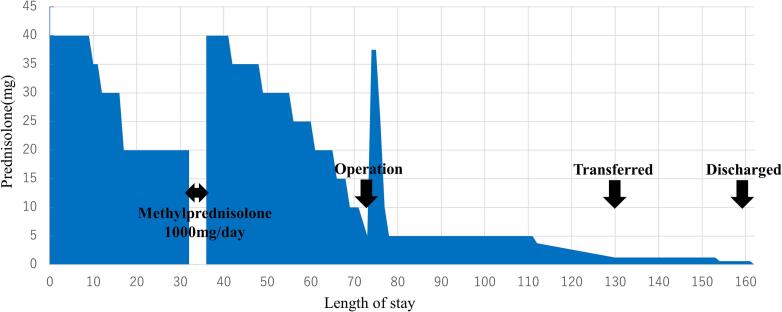
The amount of steroids administered and the clinical course.

Macroscopic resected specimen examination revealed a soft pancreas with no obvious palpable mass. A 4-mm papillary tumor was observed in the main pancreatic duct on the specimen's transected surface (Fig. 3). Histopathological examination results revealed that the papillary tumor in the main pancreatic duct had nuclear enlargement and fused growth. However, no stromal invasion was observed, and a diagnosis of IPMN with high-grade dysplasia was made (Fig. 4). An intraductal papillary mucinous adenoma (IPMA) was also observed scattered throughout the main pancreatic duct and its branches. The patient's course was uneventful, and the skin symptoms gradually improved after surgery (Fig. 1c). Steroid doses were gradually tapered on postoperative day 40 (Fig. 5), and the skin rash did not recur (Fig. 1d). On the 55th postoperative day, the patient was transferred to another hospital for rehabilitation. The patient's skin rash continued to improve, and she was discharged home on the 88th postoperative day, at which time the steroids were discontinued.

One year and four months after surgery, the patient was alive and was not receiving steroids, although scarring was present in areas where the rash had been severe.

## 3 Discussion

The term “dermadrome,” coined by Wiener, originally referred to skin symptoms reflecting visceral lesions, encompassing all skin symptoms associated with systemic diseases [2]. Various dermadromes are caused by visceral tumors, and previous reports have suggested improvements in skin lesions after tumor excision [3,4].

However, in Europe and the United States, the systematic classification and description of systemic diseases and skin symptoms based on etiology became the prevailing approach, leading to the disuse of the term dermadrome [4]. Meanwhile, in Japan, according to Mitsuhashi and Oyama, the term “dermadrome” has been used in special cases, i.e., those with “usefulness,” “unexpectedness,” and “specificity,” rather than encompassing all skin symptoms associated with systemic diseases [5,6]. Yasuda introduced the term “syndroma dermato-tumorale” for dermadromes caused by visceral or systemic malignancies [7]. Syndroma dermato-tumorale are further classified into specific and nonspecific skin lesions based on the skin lesion's nature. Dermatotumor lesions are often referred to as cutaneous metastases of visceral malignancies. They are called “specific” because the primary tumor can be specifically identified through skin lesion biopsy results. In contrast, nonspecific skin lesions are classified into groups 1 and 2. Group 1 manifests with pruritus and erythema attributed to endogenous poisoning or allergies and is believed to result from structures damaged by malignant tumors or abnormal substances produced by their influence on the surrounding tissues. Group 2 includes dermatoses, such as herpes zoster and dermatomyositis, each of which is considered an independent skin disease; these diseases have a high incidence of being associated with visceral malignancies [7].

In this case, the patient presented with generalized blistering, erythema, and lesion exacerbation. Autoimmune bullous diseases and infectious, drug-induced, and collagen diseases were the most possible causes; however, these were ruled out as the disease was refractory to treatment with steroids and immunosuppressive agents.

The patient was diagnosed with mixed-type IPMN with high-risk stigmata using EUS. The skin rash improved after pancreatic lesion resection, leading to the diagnosis of a dermadrome. The dermadrome was classified as Group 1, as described above.

In a report of eight cases of dermatomyositis associated with visceral malignancies, five patients underwent primary lesion excisions, and all showed skin rash improvement, with the duration of improvement ranging from 1 week to 1 year [3].

Necrolytic migratory erythema is conventionally associated with 70 %–90 % of glucagonomas [4]. In a report of six patients with necrolytic migratory erythema due to glucagonomas who underwent primary pancreatic lesion resections, skin rash in all patients tended to improve within 1 week postoperatively, and no recurrence was observed thereafter [4]. Similarly, in the present case, the rash gradually improved after surgery, allowing for steroid dose tapering and eventual treatment termination [4].

A search of PubMed (1950–2023) using the keywords “IPMN,” “dermadrome,” and “erythema” did not yield any relevant reports. A search in the Central Journal of Medicine (1964–2023) using the keywords “IPMN” and “dermadrome” revealed only one case report of a pancreatic tumor with necrotic migratory erythema that was preoperatively diagnosed as IPMN, but later confirmed as a nonfunctioning pancreatic neuroendocrine tumor based on histopathological findings [8].

Dermadromes caused by other pancreatic diseases, such as necrolytic migratory erythema, manifest as erythematous eruptions that spread in an annular to cricoid pattern, accompanied by erosions, blisters, and pustules. Dermadromes are conventionally associated with glucagonomas but have also been reported in liver disorders, chronic pancreatitis, heavy alcohol consumption, and eating disorders [7,9]. In many cases, low plasma amino acids, albumin, and zinc levels were observed, which may be involved in the disease pathogenesis [9]. Our patient had a similar skin rash, but there was no obvious deficiency in amino acids, albumin, or zinc. Moreover, supplementation with these substances did improve the skin rash.

Subcutaneous nodular fat necrosis, which is a cutaneous manifestation of fat necrosis caused by elevated pancreatic intraductal pressure, was first reported by Chiari [10]. This condition results from high levels of pancreatic enzymes in the blood that cause fat necrosis [11]. However, in that case, skin symptoms primarily included blisters, erosions, and erythema, and no abnormalities were observed in the subcutaneous fat on skin biopsy, which was not consistent with the present case.

The pathogenesis of dermadromes involves tumor cell growth factor-α produced by malignant tumor cells that induce epidermal cell proliferation via epidermal growth factor receptors expressed on the epidermis. Additionally, skin lesions may arise due to complex nutritional disorders occurring in conjunction with tumors, as well as enzymes leaking from organs. Although some aspects of these mechanisms are somewhat understood, the exact pathogenesis remains unknown [12]. Necrolytic migratory erythema in glucagonoma is classified as group 2; however, in this case, the absence of specific nutritional disorders complicated the understanding of the underlying mechanism.

This case was thought to have been caused by an IPMN. Although this case is unprecedented and controversial, the patient's skin rash, which had been worsening and did not improve with any treatment, including high-dose steroids, began to improve after surgery. Therefore, we believe this is the first reported case of IPMN associated with a dermadrome.

Regarding the surgical procedure, a contrast-enhancing mural nodule measuring 6 mm in the pancreatic cyst was considered a high-risk stigmata, according to the 2017 revision of the International Clinical Practice Guidelines [13], necessitating surgery. Total pancreatectomy is considered for cases wherein the main pancreatic duct is dilated over its entire length, and a wide range of mural nodules are seen within the main pancreatic duct [14]. The MRI and EUS findings in the present case were consistent with these recommendations.

The IPMN's EUS findings showed the suspected malignant nodule in the pancreatic head. Thus, pancreaticoduodenectomy was considered for resecting the nodule and preserving pancreatic function. However, if IPMA, which is assumed to exist in the main pancreatic duct and throughout the pancreas, was the skin rash's cause, we were concerned that the rash might not improve by leaving the pancreas intact. Additionally, considering the patient's advanced age, poor general condition, and the surgery being performed under high-dose steroid therapy, the risk of postoperative pancreatic fistula was deemed high.

## 4 Conclusion

In conclusion, we present a dermadrome case caused by IPMN. When encountering treatment-resistant skin rashes, a systematic search for visceral malignancies should be performed for accurate differential diagnosis.

## Consent for publication

Written informed consent was obtained from the patient for the publication of this case report and the accompanying images. A copy of the written consent form is available for review by the editor-in-chief of this journal upon request.

## Ethical approval

This study was approved by the Institutional Review Board of Gifu University Hospital.

Approval number 2023-047.

## Funding

This study did not receive any specific funding grants.

## Author contributions

MK drafted the manuscript. KM, MF, YS, YT, and NM provided academic advice. All the authors have read and approved the final version of the manuscript.

## Guarantor

Nobuhisa Matsuhashi.

## Declaration of competing interest

The authors declare that they have no conflict of interests.
